# Choice bundling, unpacked: Observed and predicted effects on intertemporal choice in an additive model of hyperbolic delay discounting

**DOI:** 10.1371/journal.pone.0259830

**Published:** 2021-11-12

**Authors:** Jeffrey S. Stein, Gregory J. Madden

**Affiliations:** 1 Center for Health Behaviors Research, Fralin Biomedical Research Institute at Virginia Tech Carilion, Roanoke, Virginia, United States of America; 2 Department of Human Nutrition, Foods, and Exercise, Virginia Tech, Blacksburg, Virginia, United States of America; 3 Department of Psychology, Utah State University, Logan, Utah, United States of America; University of Kansas, UNITED STATES

## Abstract

One method known to increase preference for larger, later rewards (LLRs) over smaller, sooner rewards (SSRs) is choice bundling, in which a single choice produces a series of repeating consequences over time. The present study examined whether effects of choice bundling on preference for LLRs: (1) increase with the number of rewards in the bundle (i.e., bundle size); (2) are independent of differences in reward magnitude between conditions; and (3) accord with predictions of an additive model of hyperbolic delay discounting, in which the value of a bundle of rewards can be expressed as the summed discounted value of all rewards in that bundle. Participants (*N* = 252) completed a choice task to assess valuation of monetary LLRs at bundle sizes of 1 (control), 3, and 9 rewards per choice (ascending/descending order counterbalanced). To control for the magnitude effect, the total reward amounts were held constant across conditions. Choice bundling significantly increased LLR preference (*p* < .001), with the largest effect observed at the largest bundle size. The descending bundle-size order produced significantly greater LLR preference than the ascending order (*p* < .05), although order did not significantly interact with bundle size. Difference scores between observed measures and those predicted by an additive model of hyperbolic discounting were small and not significantly different than zero, but were not equivalent to zero. Future research should investigate the clinical utility of choice bundling for reducing the maladaptive health behavior (e.g., substance use) with which delay discounting is associated.

## Introduction

Rewards are devalued as a function of the delay until they are received—a process known as delay discounting [1, 2]. In observational studies, delay discounting is a robust marker of maladaptive health behaviors and outcomes, including tobacco, alcohol, and other substance use [3–6]; energy intake, sedentary activity, and obesity [7–9]; and nonadherence to medications for type 2 diabetes, breast cancer, hypertension, and high cholesterol [7, 10–12]. Additional evidence suggests that delay discounting may play an etiological role in lifestyle-related disease, as interventions that decrease and increase delay discounting also improve and worsen, respectively, the health behaviors with which delay discounting is associated, including consumption or valuation of cigarettes [e.g., 13, 14], alcohol [e.g., 15], and obesogenic foods [e.g., 16–18; but also see 19, 20]. Given these considerations, understanding how delay discounting influences choice is critical and may lead to development of effective clinical interventions for health behavior.

**Fig 1:**
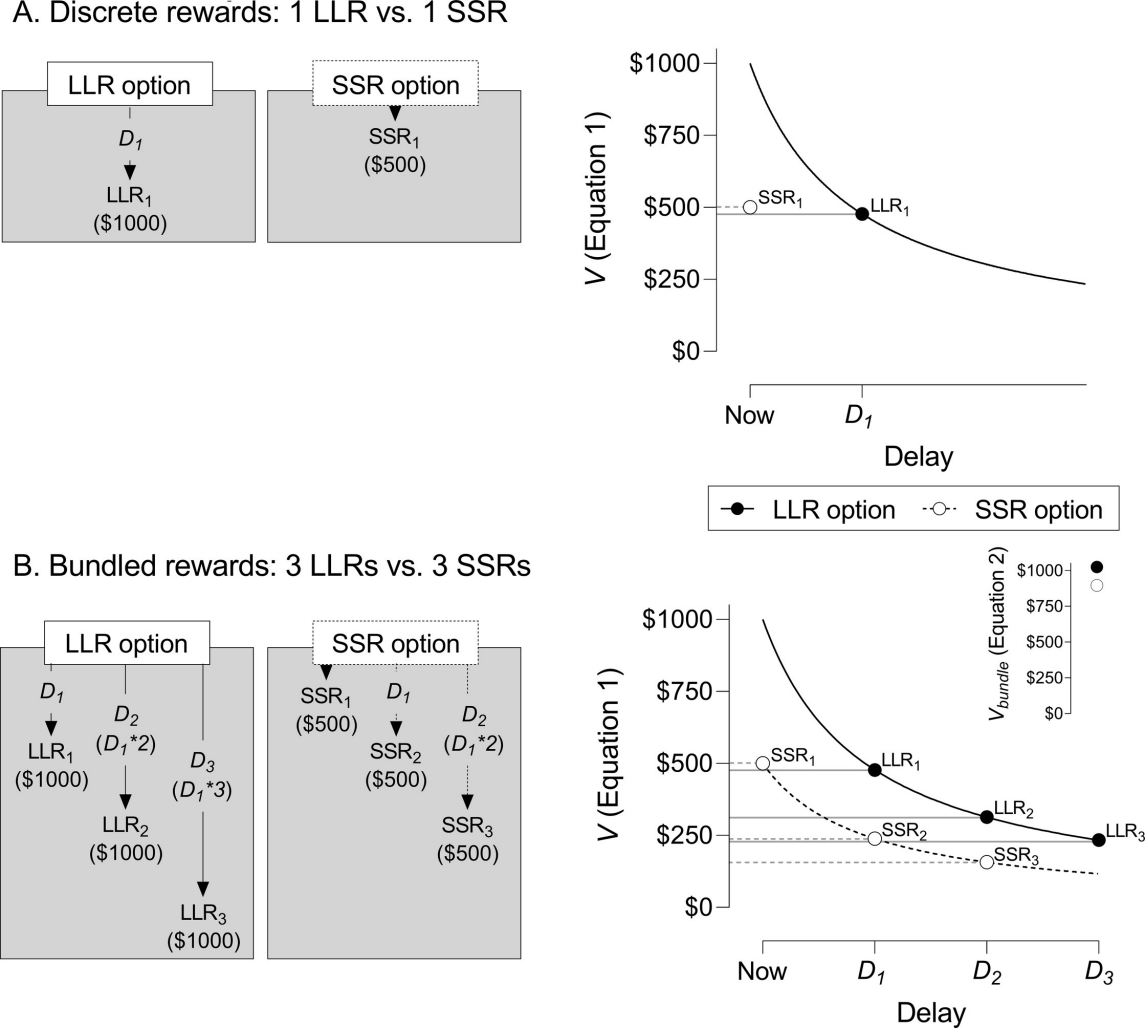
Example of predicted effects of choice bundling on preference for larger, later rewards (LLRs) over smaller, sooner rewards (SSRs). *Panel A* depicts a choice between: (1) a discrete LLR (LLR_1_) delivered after a delay (*D*_*1*_; in this example, one year); and (2) a discrete SSR (SSR_1_), delivered immediately. The corresponding graph to the right illustrates how the discounted value (*V*) of the LLR decreases hyperbolically according to Eq 1 (in this example, *k* = 0.003). Individual data points depict the subjective value of the LLR and SSR rewards. Note that the SSR value is not discounted because it is available immediately. Here, Eq 1 predicts preference for the SSR because its subjective value exceeds that of the LLR. *Panel B* depicts a choice between two bundled reward options, each comprising three rewards: (1) LLR_1_, LLR_2_, and LLR_3_ delivered after *D*_*1*_, *D*_*2*_, *and D*_*3*_, respectively; and (2) SSR_1,_ SSR_2_, and SSR_3_ delivered immediately, after *D*_*1*_ and *D*_*2*_, respectively. The graph to the right illustrates how the discounted values (*V*) of both the LLR and SSR decrease with increasing delay in accord with Eq 1 (*k* = 0.003, as in Panel A). Individual data points depict the subjective value of individual rewards in their respective bundles, with the inset depicting summed discounted values (*V*_bunde_) of these rewards according to Eq 2. Here, preference for the LLR option is now predicted because its summed value exceeds that of the SSR option.

When the subjective value of a reward is assessed across a range of delays, the nonlinear function that describes the data approximates the hyperbolic form [21]:

$$V=\frac{A}{1+kD}$$ (1)

in which *V* is the discounted value of the reward, *A* is its objective amount, *D* is its delay, and *k* is a free parameter that describes the degree of discounting. This model may be used to predict intertemporal choice, or preference between larger, later rewards (LLRs) and smaller, sooner rewards (SSRs). For example, in Fig 1A, an individual faces a choice between receiving either $1000 after a single delay (*D*_*1*_) or $500 immediately. The subjective value of the $1000 LLR is discounted proportional to the delay and the prevailing value of *k* (in this example, *k* = 0.003); however, the value of the immediate $500 SSR is undiscounted and equal to its objective, nominal value. In the choice in Fig 1A, the discounted value of the LLR at *D*_*1*_ falls below that of the undiscounted SSR; thus, Eq 1 predicts preference for the SSR option.

One method of increasing willingness to wait for LLRs, originally proposed by Ainslie [22], involves bundling behavioral consequences into a series of rewards delivered over time. In this paradigm, a single choice for the LLR or SSR option produces a series of reward deliveries after successive intervals, rather than a discrete LLR or SSR. This effect of *choice bundling* on preference for LLRs is predicted quantitatively by an extension of Eq 1 [23]:

$$V_{bundle}=\sum_{i=1}^{n}\left(\frac{A}{1+kD}\right)$$ (2)

in which the value of a bundled series of rewards (*V*_*bundle*_) is equal to the summed discounted values of all rewards in the bundle (all parameters are as described for Eq 1). A canonical example is provided in Fig 1B. Here, an individual faces a choice between a bundle of three $1000 LLRs (LLR_1_, LLR_2_, and LLR_3_) and three $500 SSRs (SSR_1_, SSR_2_, and SSR_3_). In the LLR option, the first reward is delivered after *D*_*1*_ (as in Panel A), and the second and third rewards are delivered after *D*_*2*_ and *D*_*3*_, respectively. In the SSR option, the first reward is delivered immediately (as in Panel A) and the second and third rewards are delivered after *D*_*1*_ and *D*_*2*_, respectively. Summing the discounted values of rewards in each bundle (according to Eq 2, with *k* = 0.003) yields a summed value of LLR rewards ($477.33 + $313.48 + 233.37 = $1024.18) that exceeds the summed value of SSR rewards ($500 + $238.66 + 156.74 = $895.40). Thus, Eq 2 now predicts preference for the LLR option, even though the discount rate parameter (*k*) and the relative difference in objective amounts of these options have not changed. This shift in preference is due to the hyperbolic shape of the discounting curve in which the steepness of the curve (rate of devaluation) diminishes with increasing delay, which allows the discounted values of distal LLR rewards in the bundle to accumulate in *V*_*bundle*_ at a greater rate than those of bundled SSRs [22, 24]. To illustrate, note that the subjective value of the first LLR in Fig 1B (LLR_1_) falls below that of its paired SSR (SSR_1_). However, the values of both distal LLRs (i.e., LLR_2_ and LLR_3_) in the flatter portion of the discounting curve exceed those of their paired SSRs (i.e., SSR_2_ and SSR_3_). In this way, bundling the values of distal and proximal rewards can shift preference from SSRs to LLRs.

Despite support for this additive model of hyperbolic discounting from several human and nonhuman studies [25–31; for review, see 32] at least three scientific gaps remain. First, all procedures used to date confound the choice bundling manipulation with reward magnitude. In procedures in which both the LLR and SSR options are bundled and available concurrently [25, 29–31], the bundling manipulation increases the total reward magnitude available from each option compared to a control condition with discrete rewards—even if the relative difference in magnitudes between options remains constant across conditions. Moreover, in procedures arranging bundled LLRs and a discrete SSR [26–28], the bundling manipulation increases both the total reward magnitude available from the LLR option and the relative difference in magnitude between the LLR and SSR option. This interdependence is concerning because prior literature shows that humans discount larger rewards at lower rates than smaller rewards [33, 34]; thus, this “magnitude effect” may have been mistaken, in whole or in part, for an effect of choice bundling. This potential confound is less concerning in the nonhuman studies [29, 31] because the magnitude effect has rarely been reported in nonhumans [35, but also see 36]. Nonetheless, more human research is needed using procedures that isolate the potential effects of choice bundling from those of reward magnitude.

Second, Eq 2 predicts a positive relation between bundle size (i.e., the number of sequential rewards in the bundle) and LLR preference. That is, greater willingness to wait for the LLR option should be observed with more rewards in the bundle. To date, only two studies to our knowledge have evaluated this hypothesis [28, 29] and only in nonhuman subjects. These authors found that LLR preference increased with bundle size. However, the generality of this effect in humans has yet to be examined.

Third, and finally, the quantitative accuracy of Eq 2 in predicting the effects of choice bundling remains unclear. Comparing observed effects to those predicted by the hyperbolic discounting model may provide confirmatory evidence for the proposed mechanism underlying choice bundling effects, or suggest alternative mechanisms. Two nonhuman studies [28, 29] have reported approximate concordance between observed and predicted effects, although small sample sizes limit conclusions about generality of these findings.

In the present study employing human participants, we used a within-subjects design to examine effects of choice bundling on willingness to wait for LLRs. We addressed these three gaps in knowledge by: (1) controlling for magnitude effects, (2) examining parametric effects of bundle size, and (3) comparing observed data to model predictions. In a within-subjects design, an online sample of 252 adults completed choice tasks at bundle sizes of 1 (control), 3, and 9 rewards per choice. Participants completed these three conditions in either ascending or descending order (order counterbalanced).

## Materials and methods

### Participants

Participants were recruited from Amazon Mechanical Turk, a crowdsourcing platform that allows participants to complete human intelligence tasks (HITs) for monetary compensation. To be eligible for this study, participants were required to: (1) have had previous HITs approved by requesters at least 95% of the time; (2) have completed at least 100 previous HITs; (3) reside in, and access the survey from, the United States (assessed through Amazon Payments mailing address and Qualtrics’ GeoIP Location feature, respectively); and (4) pass a “No CAPTCHA reCAPTCHA” response item [37], which estimates whether screen activity is produced by a human or a computer program (detection of the latter prevented survey continuation).

Of 286 recorded responses, 34 were ineligible (*n =* 33 had a non-US IP address and *n* = 1 did not advance beyond the CAPTCHA item). This left 252 participants who were eligible for and completed the survey. These participants required a median time of 6.28 minutes to complete the survey (interquartile range: 4.85–8.55) and received $1.50 in compensation (effective hourly wage: $14.33/h). Participants completed the study on October 11, 2020.

### Procedures

Study procedures were implemented using Qualtrics online survey software (Qualtrics, Provo, UT). All procedures were reviewed and approved by the Virginia Tech Institutional Review Board.

#### Demographics

Participants first completed a demographic questionnaire, as used previously [12, 38].

#### Intertemporal choice

Next, participants completed the five-trial, adjusting-delay task [39] to assess intertemporal choice at each of three different bundle-size conditions (1, 3, and 9). Participants were randomly assigned to complete these conditions in either ascending or descending bundle-size order. Prior to completing any of the bundle-size conditions, participants read the following instructions [adapted from those used previously; e.g., 39]

*You will now be presented with a series of choices between receiving different amounts of money at different points in time. In some questions, the amounts will be delivered all at once in lump sums. For example, you may see a question like this:*

*Which would you rather receive?*

*A. $500 now*

*or*

*B. $1000 in 3 weeks*

*In other questions, the total amounts of each choice option will be the same as those above ($500 and $1000), but these amounts will be delivered in installments over time. For example, you may see a question like this:*

*Which would you rather receive?*

*A. $166.67 now, $166.66 in 3 weeks, and $166.67 in 6 weeks*

*or*

*B. $333.33 in 3 weeks, $333.34 in 6 weeks, and 333.33 in 9 weeks?*

*These questions are hypothetical, but please choose your answer as if you will receive the money in the time frame(s) selected. Please pay close attention to the amount and time frame(s) of each option, and choose accordingly. There are no right or wrong answers in this task. Please take your time.*

**Fig 2:**
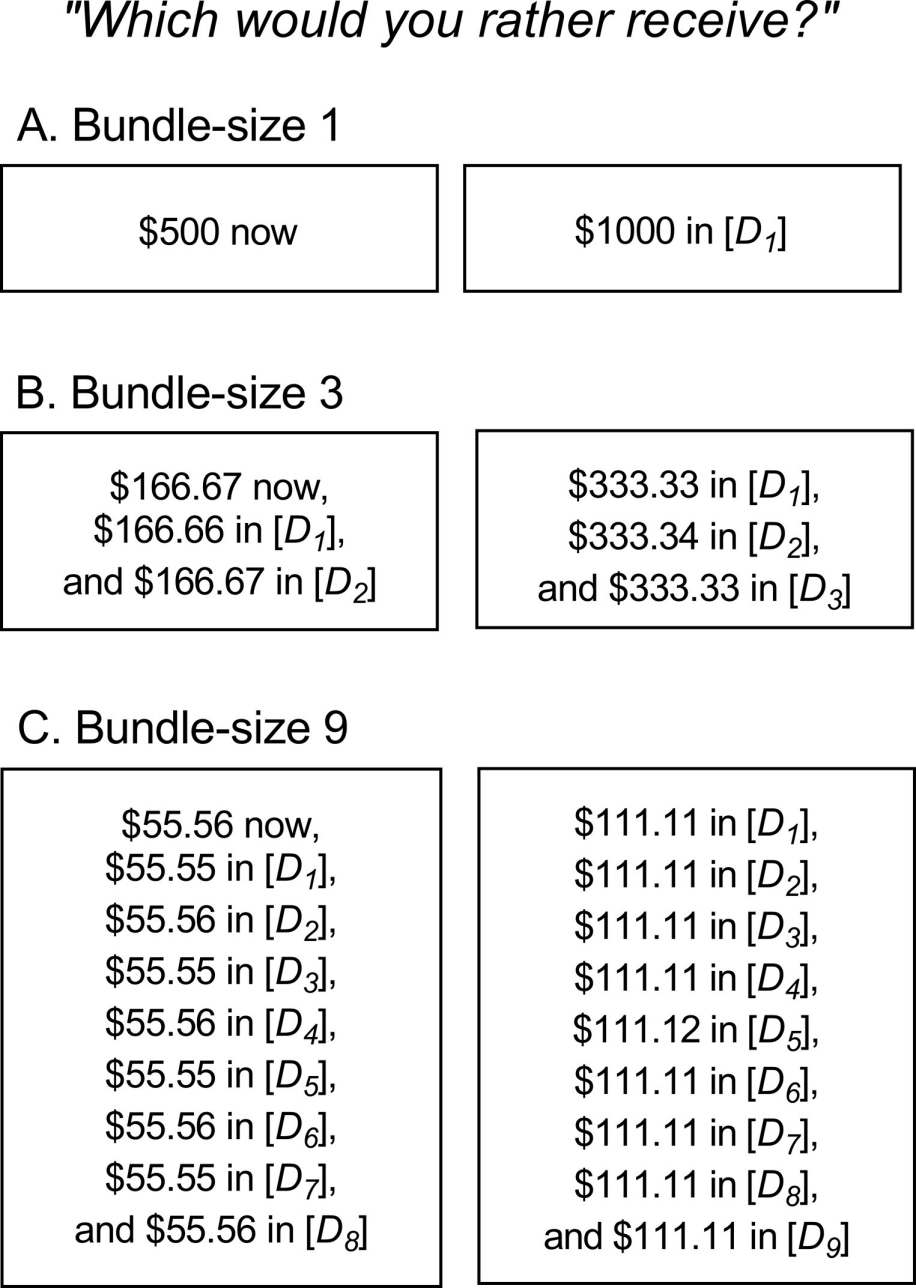
Choices presented in the bundle-size 1, 3, and 9 conditions. The delay to the first LLR in all conditions was *D*_*1*_, with additional rewards in the bundle (where applicable) delivered after *D*_*2*_, *D*_*3*_, etc. These additional delays were equal to *D*_*1*_ multiplied by the order of that delay in the series (e.g., *D*_*2*_ = *D*_1_*2, *D*_*3*_ = *D*_1_*3, etc.). The first SSR in all conditions was delivered immediately, with additional rewards in the bundle (where applicable) delivered after *D*_*1*_, *D*_*2*_, *D*_*3*_, etc. Note that with this method of arranging delays, the interval between any two rewards in a given bundle was equal to *D*_*1*_. Likewise, any given SSR in a bundle was available *D*_*1*_ units sooner than its paired LLR.

Fig 2 depicts the choices presented in each of the bundle-size conditions. The bundle-size 1 (BS1) condition was identical to the five-trial adjusting-delay task developed by Koffarnus and Bickel [39]. Here, participants made repeated, hypothetical choices between receiving either $1000 after a single delay (*D*_*1*_) or $500 immediately. The position of these LLR and SSR options on the screen (left or right) was randomized on every question. The value of *D*_*1*_ started at three weeks and adjusted after each trial based on the previous choice until reaching an indifference delay (one of 32 possible values, ranging from 1 h—25 y, in approximately logarithmic intervals), at which point the subjective values of the LLR and SSR choice options are approximately equal.

The bundle-size 3 and 9 conditions (BS3 and BS9, respectively) were identical to the BS1 condition, with the following exceptions. Both the LLR and SSR choice options comprised either 3 (BS3) or 9 (BS9) rewards delivered over time. The delay to the first LLR in both conditions was *D*_*1*_, as described above, with additional rewards in the bundle delivered after *D*_*2*_, *D*_*3*_, etc. These additional delays were equal to *D*_*1*_ multiplied by the order of that delay in the series (e.g., *D*_*2*_ = *D*_1_*2, *D*_*3*_ = *D*_1_*3, etc.; see Fig 2). The first SSR in both conditions was delivered immediately, with additional rewards in the bundle delivered after *D*_*1*_, *D*_*2*_, *D*_*3*_, etc. Note that with this method of arranging delays, the interval between any two rewards in either bundle (LLR or SSR) was equal to *D*_*1*_. Likewise, any given SSR in a bundle was available *D*_*1*_ units sooner than its paired LLR.

In these BS3 and BS9 conditions, the value of *D*_*1*_ was adjusted across trials as described for the BS1 condition, with the values of additional delays in the bundle adjusted accordingly. In order to ensure that the total amounts available from the LLR and SSR choice options remained constant across bundle sizes, the monetary amounts of individual rewards in the bundle were equal to the amounts from the BS1 condition divided by the bundle size (e.g., $1000/3 = $333.33). Individual reward amounts were adjusted in $0.01 increments to correct for rounding errors, where needed (see Fig 2).

#### Data quality

Within the survey, data quality was monitored using four quality control questions. One was embedded in the demographics questionnaire, in which participants were instructed, as follows: “Most of the options below are colors. Please select the option that is not a color.” Response options included “red”, “yellow”, “blue”, “purple”, and “winter”, with the location of the latter randomized among the other response options. Choice of any response except “winter” was interpreted as inattention. Three additional questions were specific to the choice tasks. Because the five-trial task does not allow use of standardized diagnostic criteria to assess orderliness of obtained data [e.g., 40], these quality control questions were appended to the five-trial, adjusting-delay task, as has been done previously [12, 38]. Specifically, in each of three bundle-size conditions, a sixth trial asked participants to choose between $500 now and $1000 now. In the BS3 and BS9 conditions, these monetary amounts were framed as separate rewards, as described above for these conditions; however, all delays were removed (e.g., $333.33 now, $333.34 now, and $333.33 now). Choice of the smaller option in these questions was interpreted as inattention or atypical valuation of monetary rewards.

#### Data analysis

All analyses were performed in MINITAB statistical software version 20.1 (MINITAB Inc., State College, PA). The approximate sample size was informed by a power analysis in which 260 participants provides 95% statistical power to detect at least a small (*f* = .10) within- and between-subjects factors interaction in repeated-measures ANOVA, assuming an alpha value of .05, two groups, three repeated measures, and a correlation between repeated measures of .50.

*Participant characteristics*. Participant characteristics were compared between the ascending and descending bundle-size orders using *t* tests and Fisher’s Exact tests, as appropriate.

*Dependent measures of intertemporal choice*. The dependent measure of intertemporal choice was indifference delay (in days), calculated as the adjusted value of *D*_*1*_ after the final trial of the five-trial adjusting-delay task. This is similar to measures of indifference delay used and validated previously with discrete rewards [12, 39, 41, 42]. Longer values of indifference delay reflect greater willingness to wait for LLRs. Indifference delay was used as the measure of intertemporal choice, rather than the more commonly used discount rate, *k* [21], because choice bundling was not expected to alter discount rate directly; indeed, Eq 2 assumes a constant value of *k* for all rewards. Rather, choice bundling was expected to alter the cumulative discounted values of choice options, thus increasing willingness to wait for the LLR option. Indifference values were non-normally distributed (positive skew) and were thus log transformed prior to analysis.

*Effects of bundle size on intertemporal choice*. Effects of bundle size on intertemporal choice were examined using repeated-measures ANOVA, including bundle size (1, 3, and 9) as a within-subjects factor, order (ascending and descending) as a between-subjects factor, and a Bundle Size x Order interaction term. In the primary analysis, participants who failed one or more quality control questions were excluded. In a sensitivity analysis, ANOVA was repeated when including all participants. In both analyses, ANOVA was followed by planned within-subjects comparisons between individual bundle-size conditions, and planned between-subjects comparisons between order groups at individual bundle-size conditions. Bonferroni correction was used to maintain family-wise Type 1 error rate of .05.

*Comparing model predictions to observed data*. To estimate the accuracy of Eq 2 in predicting choice with bundled rewards, observed and model-predicted data were compared. Specifically, to derive predicted indifference delays in the BS3 and BS9 conditions, we input individual participants’ *k* values [i.e., 1/indifference delay; 41] from the BS1 control condition into Eq 2 and solved for the value of *D*_*1*_ at which LLR *V*_*bundle*_ = SSR *V*_*bundle*_. The delay value in the five-trial adjusting-delay task nearest to this calculated value was the predicted indifference delay. All LLR and SSR amount parameters in these calculations were as described in Fig 2. Next, observed log indifference delays in the BS3 and BS9 conditions were subtracted from predicted values. Difference scores greater than or less than zero would indicate that Eq 2 over- or underestimated, respectively, the effects of bundling on log indifference delay. One-sample *t* tests were used to determine whether these scores differed significantly from zero.

In an exploratory analysis, one-sample equivalence tests were also used to evaluate whether difference scores were equivalent to zero [43]. Specifically, in one-sample equivalence tests, the equivalence interval was defined as zero plus or minus 0.1 standard deviations in difference scores, considered a negligible effect size [44]. The one-sample equivalence test evaluates the null hypothesis that observed data are not equivalent to zero (i.e., are ≤ or ≥ the lower and upper bounds, respectively, of the equivalence interval) against the alternative hypothesis that data are equivalent to zero (i.e., are within the lower and upper bounds). In the case of significant equivalence, the 95% confidence interval of observed difference scores would fall entirely within the equivalence interval.

These one-sample *t* tests and equivalence tests were repeated twice: once in a primary analysis in which participants who failed one or more quality control questions were excluded, and once in a sensitivity analysis in which all participants were included.

## Results

### Participant characteristics

Table 1 provides demographic characteristics for all participants, as well as those assigned to the ascending and descending bundle-size orders. No significant differences were observed between groups in any characteristic.

**Table 1:** Participant characteristics.

		Bundle-size order		
	All participants	Ascending	Descending	*p*
*n*	252	125	127	—
Age (y; ±SD)	38.02 (11.42)	38.93 (12.19)	37.13 (10.59)	.213
Gender				
% Male (*n*)	64.68 (163)	59.20 (74)	70.08 (89)	.137
% Female (*n*)	34.13 (86)	40.00 (50)	28.35 (36)	
% Different identity (*n*)	1.19 (3)	0.80 (1)	1.58 (2)	
Race				
% White (*n*)	80.16 (202)	83.20 (104)	77.17 (98)	.339
% Asian (*n*)	11.11 (28)	8.80 (11)	13.39 (17)	
% Black/African American (*n*)	3.97 (10)	2.40 (3)	5.51 (7)	
% Other race or multi-racial (*n*)	4.76 (12)	5.60 (7)	3.94 (5)	
Ethnicity				
% Hispanic/Latino	9.52 (24)	9.60 (12)	9.45 (12)	.967
Median household income, in thousands (USD; ±IQR)	55 (35, 88)	55 (35, 95)	55 (35, 85)	.966
Education				
% ≤ Some college (*n*)	36.90 (93)	40.80 (51)	33.07 (42)	.204
% ≥ 4-yr college degree (*n*)	63.09 (159)	59.20 (74)	66.93 (85)	

### Data quality

No participants failed the first quality control question (“Please select the option that is not a color.”). Thirty of 252 participants (11.90% of the sample) failed one or more of the three discounting-specific quality control questions. These numbers did not differ significantly between order groups (*n* = 14 of 125 and 16 of 127 in the ascending and descending groups, respectively; *p* = .846).

### Effects of bundle size on intertemporal choice

**Fig 3:**
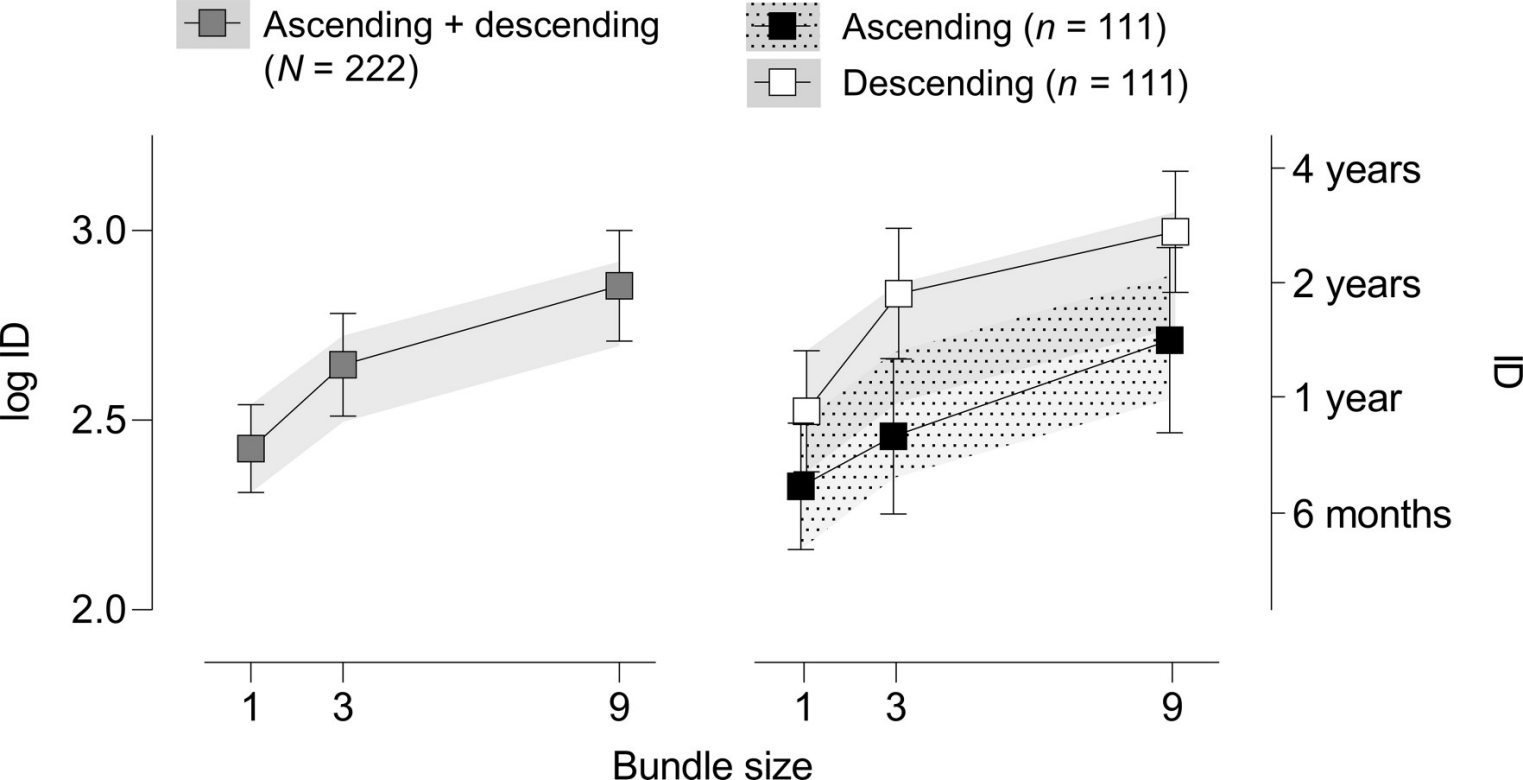
Mean log indifference delay (ID; ±95% confidence intervals) as a function of bundle size in both ascending and descending order groups combined (left panel) and in individual order groups (right panel). Gray and patterned bands represent 95% confidence intervals around model-predicted effects of bundle size, based on participants’ BS1 indifference delay values (control condition). Linear indifference delay values are scaled on the right *y* axis to aid in interpretation.

Fig 3 depicts effects of bundle size on log indifference delay. When excluding the 30 participants who failed one or more quality control questions, results of ANOVA revealed significant main effects of bundle size, *F*(2, 440) = 25.281, *p* < .001, *η*_p_^2^ = .103, and order, *F*(1, 220) = 6.30, *p* = .013, *η*_p_^2^ = .028. The Bundle Size x Order interaction was not significant, *F*(2, 440) = 1.087, *p* = .338, *η*_p_^2^ = .005. In the left panel of Fig 3 (ascending and descending orders combined), planned within-subject comparisons revealed significantly greater log indifference delays in the BS3 compared to the BS1 condition (*p* < .001; mean difference = 0.221), in the BS9 compared to the BS1 condition (*p* < .001, mean difference = 0.429) and in the BS9 compared to the BS3 condition (*p* = .002, mean difference = 0.208). In the right panel of Fig 3 (ascending vs. descending orders), planned between-subjects comparisons revealed significantly greater log indifference delays in the descending group in the BS3 condition compared to the ascending group (*p* = .015, mean difference = 0.376), with no other significant group differences at BS1 or BS9 conditions. Within-subject differences between bundle-size conditions were not examined separately in ascending and descending order groups due to the absence of a significant Bundle Size x Order interaction (described above).

In a sensitivity analysis, the repeated-measures ANOVA described above was repeated when including the additional 30 participants who failed one or more quality control questions (see S1 Fig). This analysis revealed identical conclusions as the primary analysis described above. See S1 Appendix for more details.

### Comparing model predictions to observed data

**Fig 4:**
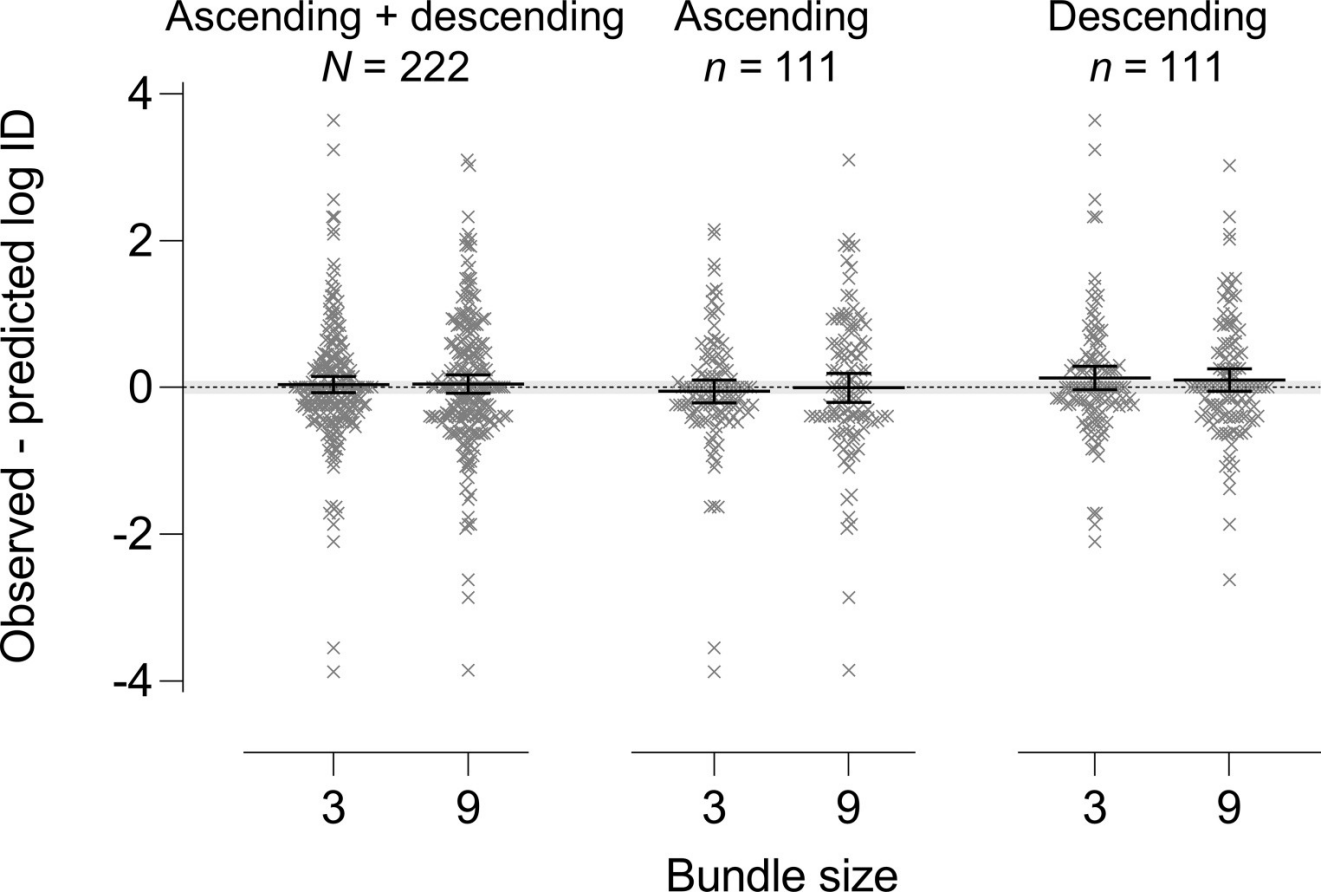
Mean difference scores (±95% confidence intervals) between observed and predicted log indifference delay (ID). Depicted are data for the BS 3 and 9 conditions (based on BS1 values) in both order groups combined (left panel), ascending order group (middle panel), and descending order group (right panel). The horizontal gray band reflects the equivalence interval, defined as zero plus or minus 0.1 standard deviations of difference scores.

Fig 4 depicts difference scores between observed log indifference delays and those predicted by Eq 2. In one-sample *t* tests excluding the 30 participants who failed one or more quality control questions, these difference scores did not differ significantly from zero in the BS3 condition when examining either: (a) the ascending and descending orders combined, *t*(221) = 0.660, *p* = .510 (mean difference = 0.037; ±0.843 SD); (b) the ascending order group, *t*(110) = 0.704, *p* = .483 (mean difference = -0.055; ±0.821 SD); or (c) the descending order group, *t*(110) = 1.592, *p* = .114 (mean difference = 0.130; ±0.857 SD). Likewise, in the BS9 condition, difference scores did not differ significantly from zero when examining either (a) the ascending and descending orders combined, *t*(221) = 0.163, *p* = .871 (mean difference = 0.047; ±0.951 SD); (b) the ascending order group, *t*(110) = 0.068, *p* = .946 (mean difference = -0.007; ±1.06 SD); or (c) the descending order group, *t*(110) = 1.293, *p* = .199 (mean difference = 0.101; ±0.823 SD).

Although observed difference scores were not significantly different from zero, results of one-sample equivalence tests indicated that the difference between predicted and obtained indifference delays were not equivalent to zero in either condition (BS3 or BS9) for the ascending and descending orders combined, ascending order group, or descending order group. In all cases, the 95% confidence intervals were not entirely confined within the equivalence interval and the mean difference values were not significantly greater than the lower equivalence limit, lower than the upper equivalence limit, or both, in any test (in all cases, p > .170).

In sensitivity analyses, the one-sample *t* tests and equivalence tests above were repeated when including the additional 30 participants who failed one or more quality control questions (see S2 Fig). These analyses revealed identical conclusions as the primary analyses described above. See S1 Appendix for more details.

## Discussion

In the largest study of choice bundling to date, bundled rewards parametrically increased willingness to wait for LLRs (i.e., increased indifference delays) in an adult, online sample of 252 participants. To contextualize these effects, the mean log indifference delay in the control condition (BS1) corresponded to a linear value of 266.11 days, or 8.75 months. In contrast, these indifference delay values were 442.85 days (14.56 months) and 715.30 days (23.51 months) per reward in the BS3 and BS9 conditions, respectively.

These observed effects of bundling on indifference delays were not due to the magnitude effect [33], as the total amounts available from the LLR and SSR options were held constant at $1000 and $500, respectively, across increasing bundle sizes. A significant main effect of order was observed, with descending bundle size producing longer indifference delays than ascending bundle size. However, the effects of choice bundling did not depend on order. Finally, the present study’s findings were partially consistent with predictions of an additive model of hyperbolic discounting (Eq 2). That is, absolute mean differences between observed and predicted log indifference delays were small (0.007–0.130 log units across condition) and did not differ significantly from zero in either primary or sensitivity analyses. However, these difference scores were not equivalent to zero.

### Choice bundling and additive hyperbolic delay discounting

Observed effects of choice bundling in the present study approximated those predicted by Eq 2. However, in an exploratory analysis, observed effects were not statistically equivalent to model predictions, which deserves consideration. Mean effects were most similar to model predictions in the ascending order group (absolute observed—predicted log ID = 0.007–0.056; see Fig 4) and least similar in the descending order group (observed—predicted log ID = 0.101–0.130). This discrepancy is consistent with the significant effect of order (see Fig 3), in which the descending order produced longer indifference delays than the ascending order. However, conclusions of one-sample *t* tests and equivalence tests were identical across both order groups; that is, mean difference scores between observed and predicted effects were not significantly different from zero, but were also not significantly equivalent to zero in either group. Thus, order effects alone were not responsible for the failure to observe equivalence with model predictions. Rather, the large heterogeneity across participants in bundling effects (0.900 pooled standard deviation in difference scores), may have limited the ability to observe equivalence. Specifically, in one-sample equivalence tests, conclusions of statistical equivalence become less likely as between-subject variance in observed effects increases, regardless of the nominal mean data values. We note also that the present study’s sample size was not powered specifically for this exploratory equivalence analysis; rather, the obtained sample size provided 95% statistical power to detect an effect of bundle size in repeated measures ANOVA. Thus, conclusions regarding equivalence between observed and predicted effects must remain preliminary.

#### Potential limitations and future directions

Investigating potential moderators of bundling effects in future research may help to explain observed heterogeneity. For example, studies may be designed to examine interactions between choice bundling and various behavioral, psychological, and sociodemographic factors that are commonly associated with delay discounting [e.g., cigarette smoking; 38, 45] or moderate effects of interventions that reduce delay discounting [e.g., working memory; 20, 46]. The present study had limited statistical power to conduct such analyses because recruitment of a general sample yields relatively few cigarette smokers or members of other clinical populations. Likewise, sociodemographic diversity in this online sample was limited, with college-educated adults overrepresented and minorities underrepresented compared to prevalence in the general U.S. population [47].

Additional research may instead seek to reduce heterogeneity in choice bundling effects by using behavioral tasks that provide greater resolution in measurement. Specifically, the five-trial adjusting-delay task used here allows measurement of only 32 possible indifference delay values, ranging from 1 h to 25 years in approximately logarithmic intervals. In future research, use of the adjusting-amount [48] or other tasks that offer more granularity may increase precision of measurement, reduce heterogeneity, and facilitate comparisons of observed bundling effects to model-predicted values. Nonetheless, the five-trial task was used in the present study because it provides rapid and generally accurate estimates of choice, thus facilitating the use of statistically powerful within-subjects designs in brief online studies. Estimates of discounting from this task also show strong concordance with those from the more common, but also more time-consuming, adjusting-amount task (e.g., r=.67-.86 for monetary rewards; [20, 39, 49]). Moreover, both tasks are sensitive to the same experimental manipulations [12, 20, 39, 50, 51] and cross-sectional associations with maladaptive health behavior [12, 38, 52].

Use of alternative behavioral tasks, however, would allow determination of whether other delay discounting models [52–55] would more precisely predict observed effects. The five-trial adjusting-delay task used here generates only a single value of indifference delay, from which estimates of *k* from the hyperbolic model can be derived [39]. Use of this task prohibits examination of quasi-hyperbolic models that add a scaling parameter (*s*) to Eq 1 [54, 56], which would require methods of assessment that allow application of nonlinear regression to estimates of discounted value across a range of delays. Nonetheless, the simple hyperbolic model used here tends to account for the most variance in observed data with the fewest free parameters [42, 57–59], which supports its application in the present study. We note, however, that predicted effects of choice bundling are theoretically unique to hyperbolic or hyperboloid models of discounting [22, 24].

A final limitation regarding use of the five-trial task in the present study is that unrealistic delays are possible at large bundle sizes, particularly in the BS9 condition. For example, the two longest possible delays on the final trial in this task are 12 years and 25 years. When multiplied by the total number of delayed rewards in the BS9 condition, these would produce delays to the final reward of 108 and 225 years, respectively, in the LLR option; and 96 and 200 years, respectively, in the SSR option. Thus, the more distal rewards in the chosen bundle may exceed participants’ expected lifespan and impact choice in an unpredictable manner. Despite this potential limitation, we note that these unrealistic delays (if contacted) are determined by participants’ own choices and would appear simultaneously for the LLR and SSR options; therefore, they are unlikely to systematically influence choice for one option over the other. Moreover, we note that the mean log indifference delay in the BS9 condition (2.85) corresponded to a raw value of 715.30 days (1.96 years), producing a realistic maximum delay to the final reward of 17.64 years. Most importantly, we note that the BS3 condition is far less prone to unrealistic delays, with maximum delays to the final reward of 36 years and 75 years. In post-hoc tests, choice bundling in this condition significantly increased indifference delays compared to the BS1 control condition, which mitigates concern over unrealistic delays in the BS9 condition. Nonetheless, in future studies using the five-trial task to manipulate choice bundling, we recommend limiting the bundle size to 3 or fewer rewards. This should not pose a substantial restriction, as both the predicted and observed relationships between bundle size and indifference delay are negatively accelerating functions (see Fig 3), with the largest incremental effects at small bundle sizes.

#### Potential clinical utility of choice bundling

Given the robust covariance between delay discounting and maladaptive health behavior [e.g., 3, 4, 60], identifying and developing methods to reduce delay discounting and increase preference for LLRs may provide therapeutic benefit in the treatment and prevention of lifestyle-related disease. That is, as many have suggested, a bias for immediate gratification may play an etiological role in lifestyle-related disease through a mechanism in which the reinforcing outcomes of many maladaptive health behaviors (e.g., cigarette smoking, consumption of obesogenic foods) are immediate and are therefore more highly valued than more beneficial, but delayed, health outcomes [e.g., avoidance of lung cancer, type 2 diabetes; 3, 7, 61, 62]. Thus, interventions that reduce bias for immediate gratification may improve health behavior.

In clinical interventions, the magnitude of observed shifts in willingness to wait may be sufficient to increase the value of long-term health consequences and improve health behavior. However, observed effects in the present and prior studies required manipulation of the timing of behavioral consequences. In contrast, most clinical interventions (e.g., for the prevention and treatment of obesity) cannot directly manipulate the consequences of health behavior in this way. For example, a treatment provider cannot control when and how often individuals at risk for diabetes will experience the health benefits of diet and exercise. As a result, adaptation of laboratory choice procedures for clinical use is required. In this regard, framing naturally occurring rewards as a bundled series of choices and consequences may hold clinical promise. For example, in addition to a condition in which the consequences of choices were bundled programmatically (as in the present study), Hofmeyr et al. [30] also showed that merely suggesting to cigarette smokers that their current choices were predictive of future choices significantly increased preference for monetary LLRs. Kirby and Guastello [25] replicated the significant effect of the suggested condition on choice for food rewards, although this effect for monetary rewards fell short of significance. These effects entail the bundling of not just consequences (as in the present study), but individual choices into a temporally extended pattern (bundle) of behavior [63].

Future research might build on these prior and present findings by developing framing or motivational interventions that emphasize that the outcomes of maladaptive health behavior are not discrete, but are chronic (and therefore bundled) conditions in which negative consequences are experienced over time (e.g., COPD, type 2 diabetes). For example, with every urge to smoke, individuals could be guided using ecological momentary intervention [64] to evaluate the bundled value of sustained, long-term good health against the value of continuing to smoke cigarettes.

Such attempts to adapt choice bundling for clinical use, however, may first benefit from a more thorough understanding of its effects in more controlled laboratory studies, particularly in clinical samples (e.g., smokers, overweight/obese participants). For example, as mentioned previously, identification of moderators of choice bundling effects may provide the ability to identify subpopulations likely to show optimal and suboptimal responses. Moreover, in order to be effective in clinical settings, choice bundling should be able to exert its effects across a wide range of choice contexts. Thus, examination of the effects of bundling on choice for monetary losses (as opposed to gains, in the present study), health gains and losses, and other commodities may provide insight into the generality or specificity of observed effects [for review, see 32]. Here, we note that the predictions of Eq 2 remain consistent regardless of the outcome examined (e.g., money, health) or its sign (gain vs. loss); however, future research is necessary in order to evaluate these predictions.

## Conclusions

We conclude that choice bundling parametrically increases willingness to wait for monetary LLRs over SSRs when controlling for differences in reward magnitude between conditions. Observed effects approximate, but are not equivalent to, those predicted by an additive model of hyperbolic delay discounting. Future research should be designed to identify predictors of heterogeneity in observed bundling effects, use more precise methods of measurement that may reduce heterogeneity, and evaluate the relative accuracy of alternative delay discounting models in predicting choice. Future research should also further explore the potential clinical utility of choice bundling, including examinations of its effects on intertemporal choice for health outcomes.

## Supporting information

S1 Fig Mean log indifference delay (ID; ±95% confidence intervals) as a function of bundle size in all participants, including n = 30 who were excluded for failing one or more quality control questions. The left panel depicts ascending and descending order groups combined and the right panel depicts individual order groups (right panel). Gray and patterned bands represent 95% confidence intervals around model-predicted effects of bundle size, based on participants’ BS1 indifference delay values (control condition). Linear indifference delay values are scaled on the right *y* axis to aid in interpretation.
(TIF)

S2 Fig Mean difference scores (±95% confidence intervals) between observed and predicted log indifference delay (ID) for the bundle-size 3 and 9 conditions (based on BS1 values) in all participants, including the n = 30 who failed one or more quality control questions. The left panel depicts both order groups combined, the middle panel depicts the ascending order group, and the right panel depicts the descending order group. The horizontal gray band reflects the equivalence interval, defined as zero plus or minus 0.1 standard deviations of difference scores.
(TIF)

S1 Appendix Description of sensitivity analysis results. (DOCX)

## References

[1] Madden GJ, Johnson PS. A delay-discounting primer. American Psychological Association; 2010. doi: 10.1037/12069-001

[2] Odum AL. Delay discounting: trait variable? Behav Processes. 2011;87: 1–9. doi: 10.1016/j.beproc.2011.02.007 21385637 PMC3266171

[3] Bickel WK, Jarmolowicz DP, Mueller ET, Koffarnus MN, Gatchalian KM. Excessive discounting of delayed reinforcers as a trans-disease process contributing to addiction and other disease-related vulnerabilities: emerging evidence. Pharmacol Ther. 2012;134: 287–297. doi: 10.1016/j.pharmthera.2012.02.004 22387232 PMC3329584

[4] MacKillop J, Amlung MT, Few LR, Ray LA, Sweet LH, Munafò MR. Delayed reward discounting and addictive behavior: a meta-analysis. Psychopharmacology. 2011;216: 305–321. doi: 10.1007/s00213-011-2229-0 21373791 PMC3201846

[5] Perry JL, Larson EB, German JP, Madden GJ, Carroll ME. Impulsivity (delay discounting) as a predictor of acquisition of IV cocaine self-administration in female rats. Psychopharmacology. 2005;178: 193–201. doi: 10.1007/s00213-004-1994-4 15338104

[6] Audrain-McGovern J, Rodriguez D, Epstein LH, Cuevas J, Rodgers K, Wileyto EP. Does delay discounting play an etiological role in smoking or is it a consequence of smoking? Drug Alcohol Depend. 2009;103: 99–106. doi: 10.1016/j.drugalcdep.2008.12.019 19443136 PMC2743449

[7] Epstein LH, Paluch RA, Stein JS, Quattrin T, Mastrandrea LD, Bree KA, et al. Delay Discounting, Glycemic Regulation and Health Behaviors in Adults with Prediabetes. Behav Med. 2020; 1–11. doi: 10.1080/08964289.2020.1712581 32275202 PMC8462992

[8] Garza KB, Ding M, Owensby JK, Zizza CA. Impulsivity and Fast-Food Consumption: A Cross-Sectional Study among Working Adults. J Acad Nutr Diet. 2016;116: 61–68. doi: 10.1016/j.jand.2015.05.003 26095434

[9] Amlung M, Petker T, Jackson J, Balodis I. Steep discounting of delayed monetary and food rewards in obesity: a meta-analysis. Psychological. 2016. Available: http://journals.cambridge.org/abstract_S0033291716000866 doi: 10.1017/S0033291716000866 27299672

[10] Reach G, Michault A, Bihan H, Paulino C, Cohen R, Le Clésiau H. Patients’ impatience is an independent determinant of poor diabetes control. Diabetes Metab. 2011;37: 497–504. doi: 10.1016/j.diabet.2011.03.004 21550831

[11] Lebeau G, Consoli SM, Le Bouc R, Sola-Gazagnes A, Hartemann A, Simon D, et al. Delay discounting of gains and losses, glycemic control and therapeutic adherence in type 2 diabetes. Behav Processes. 2016. doi: 10.1016/j.beproc.2016.09.006 27663668

[12] Vaughn JE, Ammermann C, Lustberg MB, Bickel WK, Stein JS. Delay discounting and adjuvant endocrine therapy adherence in hormone receptor-positive breast cancer. Health Psychol. 2021;40: 398–407. doi: 10.1037/hea0001077 34323542

[13] Athamneh LN, Stein JS, Bickel WK. Narrative theory III: Evolutionary narratives addressing mating motives change discounting and tobacco valuation. Exp Clin Psychopharmacol. 2019. doi: 10.1037/pha0000315 31424235 PMC7028457

[14] Stein JS, Wilson AG, Koffarnus MN, Daniel TO, Epstein LH, Bickel WK. Unstuck in time: episodic future thinking reduces delay discounting and cigarette smoking. Psychopharmacology. 2016. doi: 10.1007/s00213-016-4410-y 27553824 PMC9812225

[15] Snider SE, LaConte SM, Bickel WK. Episodic Future Thinking: Expansion of the Temporal Window in Individuals with Alcohol Dependence. Alcohol Clin Exp Res. 2016;40: 1558–1566. doi: 10.1111/acer.13112 27246691 PMC5497459

[16] Daniel TO, Stanton CM, Epstein LH. The future is now: reducing impulsivity and energy intake using episodic future thinking. Psychol Sci. 2013;24: 2339–2342. doi: 10.1177/0956797613488780 24022653 PMC4049444

[17] Sze YY, Stein JS, Bickel WK, Paluch RA, Epstein LH. Bleak Present, Bright Future: Online Episodic Future Thinking, Scarcity, Delay Discounting, and Food Demand. Clin Psychol Sci. 2017;5: 683–697. doi: 10.1177/2167702617696511 28966885 PMC5616131

[18] Hollis-Hansen K, Seidman J, O’Donnell S, Epstein LH. Episodic future thinking and grocery shopping online. Appetite. 2019;133: 1–9. doi: 10.1016/j.appet.2018.10.019 30342066 PMC6312505

[19] Stein JS, Craft WH, Paluch RA, Gatchalian KM, Greenawald MH, Quattrin T, et al. Bleak present, bright future: II. Combined effects of episodic future thinking and scarcity on delay discounting in adults at risk for type 2 diabetes. J Behav Med. 2020. doi: 10.1007/s10865-020-00178-7 32989616 PMC7965228

[20] Bickel WK, Stein JS, Paluch RA, Mellis AM, Athamneh LN, Quattrin T, et al. Does Episodic Future Thinking Repair Immediacy Bias at Home and in the Laboratory in Patients With Prediabetes? Psychosom Med. 2020;82: 699–707. doi: 10.1097/PSY.0000000000000841 32868537 PMC9664964

[21] Mazur JE. An adjusting procedure for studying delayed reinforcement. Commons, ML; Mazur, JE; Nevin, JA. 1987; 55–73. Available: https://books.google.com/books?hl=en&lr=&id=1q5mAgAAQBAJ&oi=fnd&pg=PA55&dq=mazur+adjusting+procedure&ots=eMpEXNAa1z&sig=p5vwFQCta8WlPwJmCy6lDNMqxdI

[22] Ainslie G. Specious reward: a behavioral theory of impulsiveness and impulse control. Psychol Bull. 1975;82: 463–496. Available: https://www.ncbi.nlm.nih.gov/pubmed/1099599 doi: 10.1037/h0076860 1099599

[23] Mazur JE. Theories of probabilistic reinforcement. J Exp Anal Behav. 1989;51: 87–99. doi: 10.1901/jeab.1989.51-87 2921590 PMC1338894

[24] Ainslie G. Pure hyperbolic discount curves predict “eyes open” self-control. Theory Decis. 2012;73: 3–34. Available: https://idp.springer.com/authorize/casa?redirect_uri=https://link.springer.com/content/pdf/10.1007/s11238-011-9272-5.pdf&casa_token=c8INnNrJ1asAAAAA:byFCZucLCGksRj6TrWV7-2DH9RIkHb9D8FUG5Y-lFyDximlqv9B8JytkqEHCs1EoSK2l2D5icm60cqrx

[25] Kirby KN, Guastello B. Making choices in anticipation of similar future choices can increase self-control. J Exp Psychol Appl. 2001;7: 154–164. doi: 10.1037//1076-898x.7.2.154 11477982

[26] Kirby KN. The present values of delayed rewards are approximately additive. Behav Processes. 2006;72: 273–282. doi: 10.1016/j.beproc.2006.03.011 16621330

[27] Mitchell SH, Rosenthal AJ. Effects of multiple delayed rewards on delay discounting in an adjusting amount procedure. Behav Processes. 2003;64: 273–286. doi: 10.1016/s0376-6357(03)00144-x 14580698

[28] Mazur JE. Choice between single and multiple delayed reinforcers. J Exp Anal Behav. 1986;46: 67–77. doi: 10.1901/jeab.1986.46-67 3746189 PMC1348257

[29] Stein JS, Smits RR, Johnson PS, Liston KJ, Madden GJ. EFFECTS OF REWARD BUNDLING ON MALE RATS’PREFERENCE FOR LARGER—LATER FOOD REWARDS. J Exp Anal Behav. 2013;99: 150–158. Available: https://onlinelibrary.wiley.com/doi/abs/10.1002/jeab.11?casa_token=4V0KHN4yNtgAAAAA:PFTR2Y6DCHbMMIfLIRHThwybstltRESuOyAwV9Llts_vk1Vu9QHXyaewRDZZBkgdlnfvqOBInq7Xow doi: 10.1002/jeab.11 23319442 PMC4267752

[30] Hofmeyr A, Ainslie G, Charlton R, Ross D. The relationship between addiction and reward bundling: An experiment comparing smokers and non-smokers. Addiction. 2011;106: 402–409. Available: https://onlinelibrary.wiley.com/doi/abs/10.1111/j.1360-0443.2010.03166.x?casa_token=0GPyCYBMdZQAAAAA:wMxphGp-J9GbomqO0P6C_za1ct_NjK_2OK-Yk-EJHte67pR0MCHbYet3aMxz2_hfNjJd4272EFwL8g doi: 10.1111/j.1360-0443.2010.03166.x 20955491

[31] Ainslie G, Monterosso JR. Building blocks of self-control: Increased tolerance for delay with bundled rewards. J Exp Anal Behav. 2003;79: 37–48. Available: https://onlinelibrary.wiley.com/doi/abs/10.1901/jeab.2003.79-37?casa_token=yCAH94_U84oAAAAA:UIZW2-k-d4dJAN_RCAFZt6XwrFGtISotffin8sq9fMIwnbA7NanVcfo4n6DZstrBhI_OQIp8-10evg doi: 10.1901/jeab.2003.79-37 12696740 PMC1284920

[32] Ashe ML, Wilson SJ. A brief review of choice bundling: A strategy to reduce delay discounting and bolster self-control. Addict Behav Rep. 2020;11: 100262. doi: 10.1016/j.abrep.2020.100262 32467851 PMC7244903

[33] Green L, Myerson J, McFadden E. Rate of temporal discounting decreases with amount of reward. Mem Cognit. 1997;25: 715–723. Available: https://www.ncbi.nlm.nih.gov/pubmed/9337589 doi: 10.3758/bf03211314 9337589

[34] Białaszek W, Ostaszewski P. Discounting of sequences of delayed rewards of different amounts. Behav Processes. 2012;89: 39–43. doi: 10.1016/j.beproc.2011.10.014 22062547

[35] Green L, Myerson J, Holt DD, Slevin JR, Estle SJ. Discounting of delayed food rewards in pigeons and rats: is there a magnitude effect? J Exp Anal Behav. 2004;81: 39–50. doi: 10.1901/jeab.2004.81-39 15113132 PMC1284970

[36] Grace RC, Sargisson RJ, White KG. Evidence for a magnitude effect in temporal discounting with pigeons. J Exp Psychol Anim Behav Process. 2012;38: 102–108. doi: 10.1037/a0026345 22229590

[37] Shet V. Are you a robot? introducing no captcha recaptcha. Google Security Blog. 2014;3: 12.

[38] Stein JS, Heckman BW, Pope DA, Perry ES, Fong GT, Cummings KM, et al. Delay discounting and e-cigarette use: An investigation in current, former, and never cigarette smokers. Drug Alcohol Depend. 2018;191: 165–173. doi: 10.1016/j.drugalcdep.2018.06.034 30121475 PMC6390278

[39] Koffarnus MN, Bickel WK. A 5-trial adjusting delay discounting task: accurate discount rates in less than one minute. Exp Clin Psychopharmacol. 2014;22: 222–228. doi: 10.1037/a0035973 24708144 PMC4461028

[40] Johnson MW, Bickel WK. An algorithm for identifying nonsystematic delay-discounting data. Exp Clin Psychopharmacol. 2008;16: 264–274. doi: 10.1037/1064-1297.16.3.264 18540786 PMC2765051

[41] Yoon JH, Higgins ST. Turning k on its head: comments on use of an ED50 in delay discounting research. Drug Alcohol Depend. 2008;95: 169–172. doi: 10.1016/j.drugalcdep.2007.12.011 18243583 PMC2435271

[42] Franck CT, Koffarnus MN, House LL, Bickel WK. Accurate characterization of delay discounting: a multiple model approach using approximate Bayesian model selection and a unified discounting measure. J Exp Anal Behav. 2015;103: 218–233. doi: 10.1002/jeab.128 25556903 PMC5523140

[43] De Muth JE. Tests to Identify Similarities. In: De Muth JE, editor. Practical Statistics for Pharmaceutical Analysis: With Minitab Applications. Cham: Springer International Publishing; 2019. pp. 179–196. doi: 10.1007/978-3-030-33989-0_7

[44] Cohen J. Statistical power analysis for the social sciences. 1988.

[45] Bickel WK, Odum AL, Madden GJ. Impulsivity and cigarette smoking: delay discounting in current, never, and ex-smokers. Psychopharmacology. 1999;146: 447–454. Available: http://www.ncbi.nlm.nih.gov/pubmed/10550495 doi: 10.1007/pl00005490 10550495

[46] Lin H, Epstein LH. Living in the moment: effects of time perspective and emotional valence of episodic thinking on delay discounting. Behav Neurosci. 2014;128: 12–19. doi: 10.1037/a0035705 24512061 PMC4049454

[47] U.S. Census Bureau. U.S. Census Bureau Releases New Educational Attainment Data. 2020. Available: https://www.census.gov/newsroom/press-releases/2020/educational-attainment.html#:~:text=In%202019%2C%20high%20school%20was,from%2029.9%25%20to%2036.0%25.

[48] Du W, Green L, Myerson J. Cross-cultural comparisons of discounting delayed and probabilistic rewards. Psychol Rec. 2002;52: 479. Available: http://search.proquest.com/openview/a5d28e75697efcfc24b0eb7484a335f9/1?pq-origsite=gscholar

[49] Stein JS, Sze YY, Athamneh L, Koffarnus MN, Epstein LH, Bickel WK. Think fast: rapid assessment of the effects of episodic future thinking on delay discounting in overweight/obese participants. J Behav Med. 2017; 1–7. doi: 10.1007/s10865-017-9857-8 28508382 PMC5685941

[50] Stein JS, Tegge AN, Turner JK, Bickel WK. Episodic future thinking reduces delay discounting and cigarette demand: an investigation of the good-subject effect. J Behav Med. 2017. doi: 10.1007/s10865-017-9908-1 29270887

[51] Mellis AM, Athamneh LN, Stein JS, Sze YY, Epstein LH, Bickel WK. Less is more: Negative income shock increases immediate preference in cross commodity discounting and food demand. Appetite. 2018;129: 155–161. doi: 10.1016/j.appet.2018.06.032 29959952 PMC6156798

[52] Mellis AM, Woodford AE, Stein JS, Bickel WK. A second type of magnitude effect: Reinforcer magnitude differentiates delay discounting between substance users and controls. J Exp Anal Behav. 2017;107: 151–160. doi: 10.1002/jeab.235 28101922 PMC5321101

[53] Samuelson PA. A note on measurement of utility> vol. 4, no. 2. 1937. Available: http://hermes-ir.lib.hit-u.ac.jp/da/handle/123456789/19391

[54] Myerson J, Green L. Discounting of delayed rewards: Models of individual choice. J Exp Anal Behav. 1995;64: 263–276. doi: 10.1901/jeab.1995.64-263 16812772 PMC1350137

[55] Laibson D. Golden Eggs and Hyperbolic Discounting. Q J Econ. 1997;112: 443–478. doi: 10.1162/003355397555253

[56] Rachlin H. Notes on discounting. J Exp Anal Behav. 2006;85: 425–435. doi: 10.1901/jeab.2006.85-05 16776060 PMC1459845

[57] Mazur JE, Biondi DR. Delay-amount tradeoffs in choices by pigeons and rats: Hyperbolic versus exponential discounting. J Exp Anal Behav. 2009;91: 197–211. Available: https://onlinelibrary.wiley.com/doi/abs/10.1901/jeab.2009.91-197?casa_token=m2g-YWyGvuwAAAAA:pneAMEsGFL9eNnwno_n-l4IFhRXjotmxZodXeS4yWyKD69mVQ5tHaf179pfEeUNOt8q-fFE1H-KGiw doi: 10.1901/jeab.2009.91-197 19794834 PMC2648524

[58] Green L, Myerson J. Exponential versus hyperbolic discounting of delayed outcomes: Risk and waiting time. Am Zool. 1996;36: 496–505.

[59] Madden GJ, Bickel WK, Jacobs EA. Discounting of delayed rewards in opioid-dependent outpatients: exponential or hyperbolic discounting functions? Exp Clin Psychopharmacol. 1999;7: 284–293. doi: 10.1037//1064-1297.7.3.284 10472517

[60] Snider SE, DeHart WB, Epstein LH, Bickel WK. Does delay discounting predict maladaptive health and financial behaviors in smokers? Health Psychol. 2019;38: 21–28. doi: 10.1037/hea0000695 30474996 PMC6601630

[61] Stein JS, Madden GJ. Delay Discounting and Drug Abuse: Empirical, Conceptual, and Methodological Considerations. The Wiley-Blackwell Handbook of Addiction Psychopharmacology. Wiley-Blackwell; 2013. pp. 165–208. doi: 10.1002/9781118384404.ch7

[62] Bickel WK, Snider SE, Quisenberry AJ, Stein JS, Hanlon CA. Competing neurobehavioral decision systems theory of cocaine addiction: From mechanisms to therapeutic opportunities. Prog Brain Res. 2016;223: 269–293. doi: 10.1016/bs.pbr.2015.07.009 26806781 PMC5495192

[63] Rachlin H. In defense of teleological behaviorism. Journal of Theoretical and Philosophical Psychology. 2017;37: 65–76. doi: 10.1037/teo0000060

[64] Heron KE, Smyth JM. Ecological momentary interventions: incorporating mobile technology into psychosocial and health behaviour treatments. Br J Health Psychol. 2010;15: 1–39. doi: 10.1348/135910709X466063 19646331 PMC2800172

