# Supplementary figures and images for: Choice bundling, unpacked: Observed and predicted effects on intertemporal choice in an additive model of hyperbolic delay discounting
Source: PLoS One. 2021 Nov 12;16(11):e0259830. doi: 10.1371/journal.pone.0259830 (PMC8589209; doi:10.1371/journal.pone.0259830)

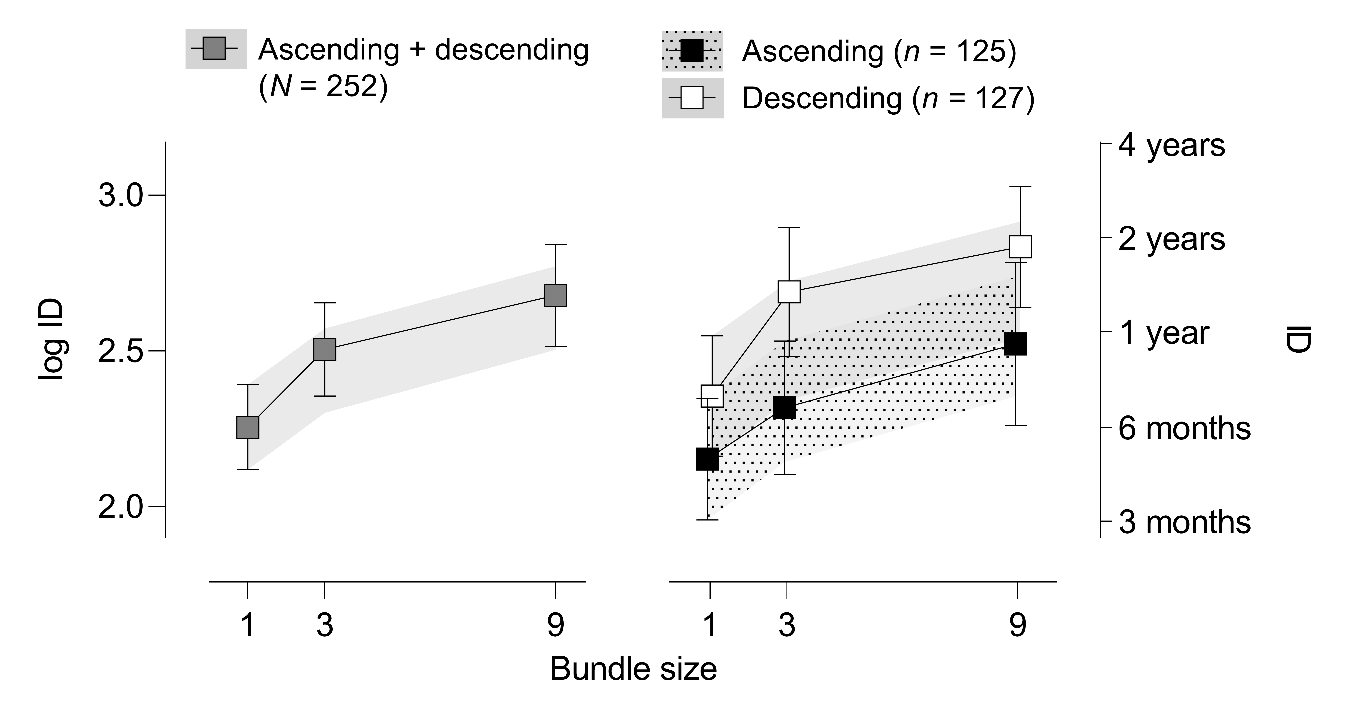

Supplement: S1 Fig — The left panel depicts ascending and descending order groups combined and the right panel depicts individual order groups (right panel). Gray and patterned bands represent 95% confidence intervals around model-predicted effects of bundle size, based on participants’ BS1 indifference delay values (control condition). Linear indifference delay values are scaled on the right y axis to aid in interpretation. (TIF) [file pone.0259830.s001.tif]

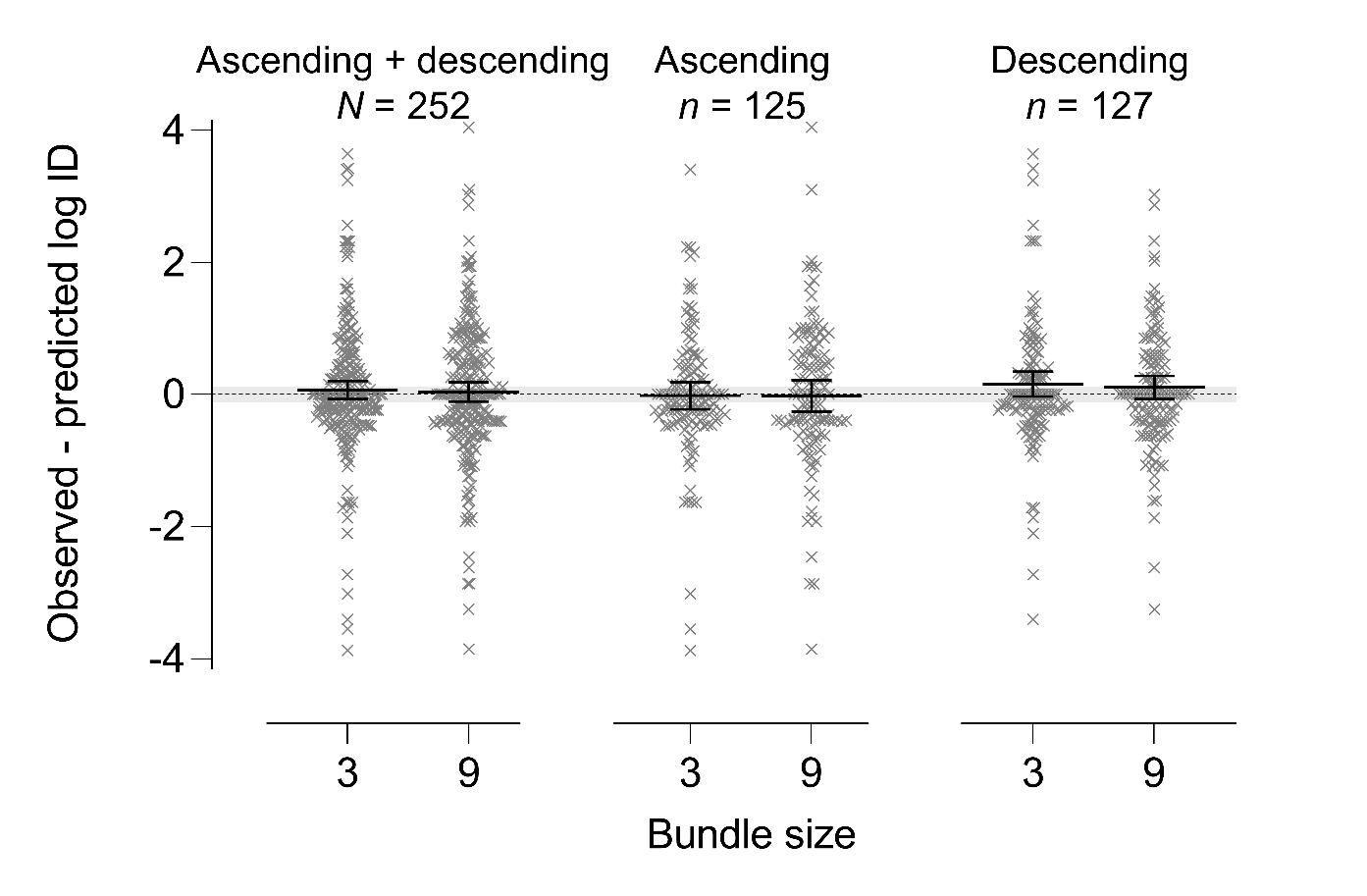

Supplement: S2 Fig — The left panel depicts both order groups combined, the middle panel depicts the ascending order group, and the right panel depicts the descending order group. The horizontal gray band reflects the equivalence interval, defined as zero plus or minus 0.1 standard deviations of difference scores. (TIF) [file pone.0259830.s002.tif]
