# Supplementary material for: Choice bundling, unpacked: Observed and predicted effects on intertemporal choice in an additive model of hyperbolic delay discounting
Source: PLoS One. 2021 Nov 12;16(11):e0259830. doi: 10.1371/journal.pone.0259830 (PMC8589209; doi:10.1371/journal.pone.0259830)
Supplement: S1 Appendix — (DOCX) [file pone.0259830.s003.docx]

# **S1 Appendix**

## **Results**

### **Effects of Bundle Size on Intertemporal Choice (Sensitivity Analysis)**

In a sensitivity analysis, ANOVA was repeated when including the additional 30 participants who failed one or more quality control questions (see Supplementary Figure 1). This analysis revealed identical conclusions as the primary analysis. That is, significant main effects of bundle size, *F*(2, 500) = 18.233, *p* < .001, *η* _p_^2^ = .068, and order, *F*(1, 250) = 5.303, *p* = .022, *η*_p_^2^ = .021 were observed, with no significant Bundle Size x Order interaction, *F*(2, 500) = 0.750, *p* = .473, *η* _p_^2^ = .003. When ascending and descending orders were combined, planned within-subjects comparisons revealed significantly greater indifference delays in the BS3 compared to BS1 condition (*p* = .001, mean difference = 0.250), the BS9 compared to the BS1 condition (*p* < .001, mean difference = 0.424), and the BS9 compared to the BS3 condition (*p* = .028, mean difference = 0.174). Likewise, when comparing ascending and descending orders, planned comparisons revealed significantly greater log indifference delays in the descending compared to ascending order group at the BS3 condition (*p* = .029, mean difference = 0.372) with no other significant group differences at BS1 or BS9 conditions.

### **Comparing Model Predictions to Observed Data (Sensitivity Analyses)**

In a sensitivity analyses, the one-sample *t* tests above were repeated when including the additional 30 participants who failed one or more quality control questions (see Supplementary Figure 2). These analyses revealed identical conclusions as the primary analyses. That is, log indifference delay difference scores did not differ significantly from zero in the BS3 condition when examining either the ascending and descending orders combined, *t*(251) = 0.978, *p* = .329 (mean difference = 0.068; ±1.110 SD), the ascending order group, *t*(124) = 0.199 , *p* = .842 (mean difference = -0.020; ±1.140 SD), or the descending order group, *t*(126) = 1.629, *p* = .106 (mean difference = 0.156; ±1.076 SD). Likewise, in the BS9 condition, difference scores did not differ significantly from zero when examining either the ascending and descending orders combined, *t*(251) = 0.557, *p* = .578 (mean difference = 0.041; ±1.178 SD), the ascending order group, *t*(124) = 0.200, *p* = .842 (mean difference = -0.024; ±1.320 SD), or the descending order group, *t*(126) = 1.161, *p* = .248 (mean difference = 0.105; ±1.019 SD).
